# Reduction in contraceptive use during the COVID-19 pandemic among women in an indigenous Mexican community: a retrospective crossover study

**DOI:** 10.3389/fpubh.2023.1189222

**Published:** 2023-09-07

**Authors:** Lilia V. Castro-Porras, María Alejandra Aguilar-Rodríguez, Mario E. Rojas-Russell, Bertha A. Salinas-Iracheta

**Affiliations:** ^1^Health, Policy and Population Research Center, National Autonomous University of Mexico, Mexico City, Mexico; ^2^Facultad de Estudios Superiores Zaragoza, UNAM, Mexico City, Mexico

**Keywords:** contraceptive, indigenous, COVID-19, women, crossover study, Mexico

## Abstract

**Background:**

Being indigenous, being a woman, and living in poverty are social determinants that contribute to reduced access to healthcare, including reproductive health services. The COVID-19 pandemic might have exacerbated this lag.

**Objective:**

This study explored how the COVID-19 pandemic affected the contraceptive use of a group of indigenous Mexican women and adolescents in their community.

**Methods:**

Between June and December of 2021, 158 indigenous Mexican women who had experienced recurrent pregnancies were interviewed at two health centers in San Cristóbal de las Casas, Chiapas. Participants were either pregnant when they completed the questionnaire or had been pregnant during the COVID-19 pandemic. Women were asked about their contraceptive practices before and during the pandemic. The change in contraceptive practice was estimated using a logistic model.

**Results:**

The COVID-19 pandemic reduced contraceptive use by 50%. Among women who wanted contraception, 58% did not receive it. During the pandemic, 77% of previous contraceptive users reported difficulty obtaining contraception, and only 23% sought family planning assistance.

**Conclusion:**

During the COVID-19 pandemic, indigenous women in the studied community used fewer contraceptive methods and did not use intrauterine devices. Additionally, there was a decline in the percentage of women using contraceptives. These results highlight the impact on indigenous populations and the difficulties they could face in accessing reproductive health services during health emergencies.

## Introduction

Human rights gaps exist among certain populations in many lower-middle and low-income nations. One such group is impoverished indigenous women, whose living conditions, low educational access, and lack of employment opportunities determine their access to healthcare (1). The reproductive health of women from indigenous communities lags behind that of other women. Various factors, such as colonization’s transgenerational negative effects, racism, low socioeconomic status, and lower education levels, have contributed to health disparities (2). Compared to other women, indigenous women have a greater gap in reproductive health (3, 4).

In Mexico, the 2020 Population and Housing Census identified 7,364,645 people aged 3 years or older who speak an indigenous language (6.1% of the total population), of whom 51.4% are women. According to the National Institute of Indigenous Peoples, there were 11,800,247 people in 2020 (9.4% of the country’s total population). If they self-identify as indigenous, the number increases to 23.2 million people aged 3 years and older (19.4% of the total population in this age range) (5, 6).

It has been reported that Mexicans and Central Americans are poor, especially women and young women of reproductive age are the poorest (7). Similar inequalities occur in other countries; for example, in Australia was reported that there was lower access to general practitioners, allied health professionals, specialists, obstetrics service providers, and pathology and diagnostic tests among indigenous women who experienced stillbirths compared with non-indigenous women (8). There is another example of Canadian Indigenous women facing numerous health and societal challenges, including domestic violence (9).

Indigenous women in Mexico have lower rates of prenatal care, have a higher fertility rate, and become sexually active at a younger age (10). Among indigenous women, most decisions related to prenatal care are made by their partners (11, 12). Indigenous adolescent women have a higher fertility rate than their non-indigenous counterparts (13).

Unforeseen situations such as the COVID-19 pandemic could deepen the gap between the health services utilization by indigenous peoples in nations like Mexico; an example of this was found in the higher risk of death from this cause, especially outside the hospital (14). In this country, during the pandemic, emergency health authorities implemented containment measures that included converting healthcare facilities into “Covid care centers,” limiting access to services that were not considered a priority during the emergency. For example, talks on the correct use of contraceptive methods or the availability of contraceptives (15).

The potential consequences on reproductive health services, particularly contraceptive methods, in indigenous communities, is one of the major concerns considering the vulnerability of indigenous populations. In this study, we employ an intersectional approach to examine the impact of the COVID-19 pandemic on the provision and use of contraceptive methods among indigenous Mexican women and adolescents. The intersectionality lens allows us to understand how various social factors, such as gender, ethnicity, and socioeconomic status, intersect and shape the experiences and vulnerabilities of this specific population. By considering the unique challenges impoverished indigenous women face, including their living conditions, limited educational access, and lack of employment opportunities, we can better comprehend the barriers they encounter in accessing reproductive healthcare. Moreover, the historical effects of colonization, systemic racism, and lower education levels contribute to health disparities in this population. By utilizing an intersectional framework, we can shed light on the complex dynamics that contribute to the reproductive health gap among indigenous women, particularly during times of crisis like the COVID-19 pandemic (16). This study aimed to explore how the COVID-19 pandemic affected the use of contraceptive methods in a group of indigenous Mexican women and adolescents.

## Methods

### Design and settings

We conducted a retrospective crossover study to investigate the impact of the COVID-19 pandemic on contraceptive use. The pandemic was regarded as a transient exposure, and the participants served as their own controls or matched pairs, transitioning between periods of varying risk, namely the pre-pandemic and pandemic periods. This design is typically used to study acute risks associated with transient exposures. The subjects were treated as matched pairs by crossing back and forth between periods of different risk (in this case: pre-pandemic and pandemic periods) (17, 18). To ensure the clarity of the survey questions used in this study, a pilot test was conducted with a sample of 27 participants. The pilot test served as an opportunity to refine the questionnaire and assess the participant’s understanding of the items. The pilot test participants were included in the final sample of the study since no problem was found with the items or the responses. We collected data on use of contraceptive methods, sociodemographic characteristics, and beliefs about the COVID-19 epidemic through interviews following a standardized questionnaire. From June to December 2021, participants with a history of at least one previous pregnancy completed the questionnaire during their visit to the health center. As a result, they were pregnant when they filled out the questionnaire or were pregnant during the COVID-19 pandemic. This aimed to compare their pregnancy experiences related to contraceptive use during and before the pandemic. They were recruited as volunteer participants at two health centers in San Cristóbal de las Casas, México. Both health centers belonged to state services of the Mexican Ministry of Health; one belonged to the first level of care while the other one was from the second level of care. These were where the health center authorities provided us with facilities for conducting the study. In a designated private room trained personnel administered the questionnaire in Spanish. Additionally, a translator proficient in Spanish and Tzotzil (the most indigenous languages spoken in San Cristóbal de las Casas community) was present on-site throughout the data collection process.

### Variables

The outcome variable was the percentage of participants who reported using contraceptives during and before the pandemic.

### Ethical considerations

The Ethics and Research Committee of the School of Medicine of the National Autonomous University of Mexico approved the protocol of the present study under Research Protocol: FM/DI/115/2019. Participation in this study was voluntary. Before completion, participants were informed of their rights as outlined in the Helsinki Declaration (19). In addition, all participants were actively informed of the study’s objective, their research rights, that there would be no consequences if they chose not to participate, and the confidential nature of their participation. This information was conveyed verbally and in writing. After that, all participants who were 18 years of age or older signed the informed consent form, indicating their agreement to participate. Participants under the age of 18, signed the informed assent form, which expressed their willingness to take part in the study, while their legal guardian or parent provided the informed consent. For illiterate women, consent or assent was obtained after ensuring their understanding of the form’s content and confirming their proficiency in Spanish. Translator assistance was available throughout the study, although it was not necessary.

### Statistical analysis

Categorical variables are expressed as frequencies and percentages (%), while continuous variables are described using means and standard deviations (SD). Age-adjusted logistic models were utilized to estimate changes in contraceptive use before and during the pandemic. The model included all factors with a value of *p* <0.05. The Hosmer-Lemeshow test was used to evaluate the model’s goodness of fit. The projected likelihood of using a contraceptive technique by age for each period was then plotted on a graph. The age intervals considered were from minimum to maximum, and between this period, we evaluated the probability every 2 years. The data were analyzed using the statistical software Stata version 15.0 (Stata Corp, College Station, Texas, United States).

## Results

158 pregnant women who had previously given birth and lived in a predominantly indigenous community participated in this study. All participants understood the Spanish language.

The average age of the participants was 21.7 years old, and 13.3% were adolescents. 8.2% of participants were illiterate, nearly 95.0% spoke an indigenous language, and 4 out of 5 women did not attend school at the moment of the study. The main reason for not attending school was lack of money (32.0%), pregnancy (18.5%), and marriage (16.0%). One in two women reported home care was their main activity during the pandemic (Table 1).

**Table 1:** Sociodemographic characteristics of participants.

Characteristic	Percentage [*n*]
Age (years)^#^	21.7 [2.1]
Young women (<20 years)	13.3 [21]
School level	
Elementary or less	40.0 [58]
Secondary	52.4 [76]
High school or more	7.6 [11]
Illiteracy	8.2 [13]
School attendance	21.6 [33]
Economic problems	57.1 [64]
Pregnancy	34.9 [37]
Marriage	31.1 [32]
Own decision	27.8 [27]
Achieving the aspired level	10.2 [10]
Family decision	7.1 [7]
School far away	5.1 [5]
Personal problems	6.1 [6]
No school or space	5.1 [3]
Health problems	3.1 [3]
Religion	3.1 [3]
COVID-19	1.0 [1]
Marriage status	
Single	0.6 [1]
Marriage	84.2 [133]
Voluntary union	15.2 [24]
Main occupation during pregnancy	
Student	1.3 [2]
Housewife	47.1 [74]
Merchant	25.5 [40]
Work for several employers	24.8 [39]
Family-owned business	0.6 [1]
Indigenous language speaking	95.6 [151]
Location of residence	
San Cristóbal de las Casas	94.3 [147]
Other	5.7 [9]

San Cristóbal de las Casas, Chiapas, México, 2021.

# Mean [Standard deviation]. Total sample = 158; Due to the non-response, some characteristics do not total to 100%.

**Figure 1:**
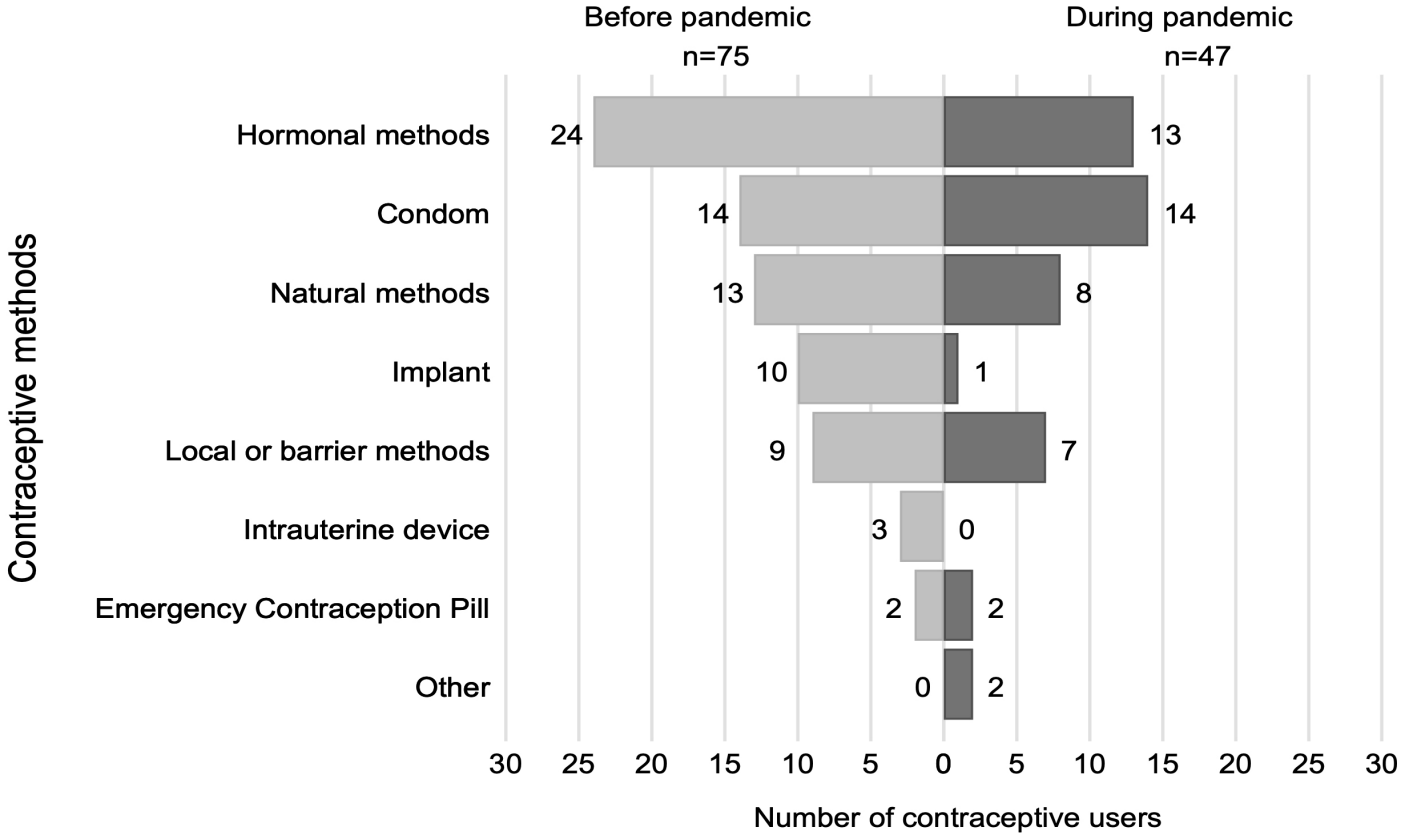
Comparison of contraceptive method use before and during the pandemic,

Almost one-third of women used contraception during the pandemic (29.7%), and nearly half used contraception before the pandemic (47.0%). During the pandemic, 77.3% of contraceptive users reported difficulties finding a method. Among contraceptive users, 79% sought family planning counseling at a health center, and of these, 73% received it (Table 2). Before the pandemic, hormonal contraception was the most common form of contraception. Whereas, during the pandemic, the condom was. The implant was the contraceptive method that showed the greatest reduction since, during the pandemic, only 1 of the 10 previous users continued to use it (Figure 1).

**Table 2:** Contraceptive use and health service utilization before and during the pandemic.

Characteristic	Percentage [*n*]
Pre-pandemic contraceptive use (Rss = 158)	47.5 [75]
During the pandemic contraceptive use (Rss = 158)	29.7 [47]
Women who could not access any contraceptive method because of the pandemic (Rss = 158)	48.1 [76]
Change of contraceptive methods during the pandemic among pre-pandemic contraceptive users (Rss = 47)	25.5 [12]
Difficulty in obtaining a contraceptive method during the pandemic among pre-pandemic contraceptive users (Rss = 75)	77.3 [58]
Family planning counseling seeking at a health center among pre-pandemic contraceptive users (Rss = 75)	78.6 [59]
Get information about contraceptives when seeking counseling (Rss = 59)	72.9 [43]

San Cristóbal de las Casas, Chiapas, México, 2021.

Rss: reference sample size.

**Figure 2:**
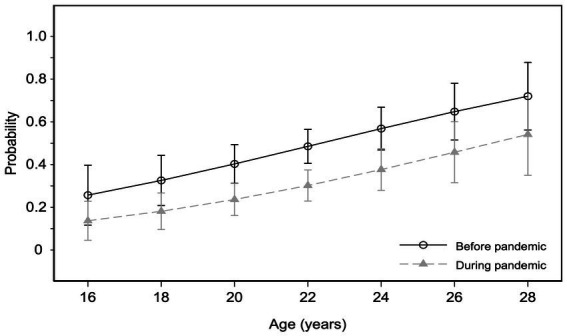
The estimated probability of contraceptive use before and during the pandemic by age.

There were more than double odds of not using any contraceptive method during the pandemic compared to the pre-pandemic period [odds ratio = 0.46 (95% CI 0.29, 0.73)] (Table 3). The plot for the probabilities resulting from the model shows that the probability of using any contraceptive method was less during the COVID-19 pandemic than in the previous period, independent of the participant’s age (Figure 2).

**Table 3:** Logistic model of using contraception*.

	OR [95% CI]	*p* value
Before pandemic	1	
During pandemic	0.46 [0.29, 0.73]	0.001

*Adjusted for age.

## Discussion

This study aimed to explore how the COVID-19 pandemic affected the use of contraceptive methods in a group of indigenous Mexican women and adolescents in their community. We found that less than half of the participants used any contraceptive method before the pandemic, and this percentage dropped to one-third during the pandemic.

The illiteracy rate in our study was 8.2% and only one in five women attended school. Lack of money (32%), pregnancy (18.5%) and marriage (16.0%) were the three main reasons why women did not attend school. In indigenous communities, marriage, community, and interpersonal relationships are institutionalized forms of gender oppression that increase indigenous women’s vulnerability and influence their decisions regarding childbirth and health care, according to qualitative research methods (1) These conditions may partially explain why our study’s participants did not attend school.

Education has a reducing effect on fertility and, above all, has an effect of delaying the timing of childbearing (20) and early pregnancy and motherhood have been strongly associated with school attendance, tertiary education, and marital or cohabitation status (21).

We found a lower percentage of participants (47%) using contraceptives before the pandemic. This result could be because the participants in our study were younger (under 28 years of age) and indigenous. Indigenous communities face significant barriers to accessing reproductive health services, which may explain the percentage of contraception use (22). Previous studies reported that lower contraceptive use in indigenous groups was associated with lower coverage ratio (23). In addition, Chiapas had the lowest percentage of sexually active women of childbearing age using contraceptive methods (63.0%); meanwhile, Mexico City reported 81.1% (24).

According to a report by the United Nations Population Fund in 2020, women with unmet contraception needs will experience a 19.6% increase in pregnancies and births resulting from the covid-19 pandemic. In this report, the analysis used economic data from Mexico and econometric models to estimate the increase (15). We found that women who could not have a method of contraception even though they wanted it was 58.3%. The small sample size could explain this result due to the small percentage of contraception users.

No significant differences were found related to being an adolescent for contraceptive use. Hubert et al. reported similar results. They analyzed the 2015 Mexican National Survey of Boys, Girls, and Women (ENIM) to explore the factors associated with pregnancies and births among young adults. According to their study, the indigenous background did not significantly influence the likelihood of an adolescent becoming pregnant. Among adult women, however, the indigenous group (87%) was significantly more likely than the non-indigenous group (64%) (25). However, our results do not agree with the reported in a previous study where the authors reported high rates of adolescent girls who did not use any form of family planning before becoming pregnant (26). Their study included only urban residents, which may explain the differences.

Concerning the type of contraceptive method used, a secondary analysis of data from the National Health and Nutrition Survey (Ensanut) for the periods 2012 and 2018–19 found, in the adolescent population, an increase in the use of long-acting reversible contraceptives (ARAP), among which are the intrauterine device and the contraceptive implant, in addition to condoms being the method most used by adolescents (21). In contrast, in the present study, among the methods least used by the participants are ARAP, with the intrauterine device being the least used and hormonal methods being the most used before and during the pandemic. This may be because the sample included people of different ages and most participants said it was hard to get a method during the pandemic. This is consistent with a study on the lack of ARAP availability in first-level units, provider-user relationships, and user information during the COVID-19 pandemic (27). Darney et al. analyzed data from three waves of a nationally representative, population-based survey to describe contraceptive use and education among rural adolescents (15–19) and young women (20–24) in Mexico. According to their study, adolescent and young adult women with high educational attainment, positive marital status, pregnancy experiences, and health insurance use modern contraceptives (28). According to recent research, indigenous women had a lower coverage ratio for modern contraception than non-indigenous women (CR: 0:73; CI 0:65–0:83) (3).

In addition, it was found that more than 70% of the sample reported difficulty in obtaining a contraceptive method. Based on previous epidemics, such as Ebola in 2014 (29) and Zika in 2015 (30), it has been documented that people stop attending health services even when needed due to fear of contracting the disease. In addition, health services may present limitations because they focus exclusively on care for patients infected with the virus. These data show the importance of identifying moderating variables for the use of contraceptive methods in healthcare situations, especially in indigenous populations whose demographic and social characteristics make them at-risk groups.

It has been found that 89.3% of indigenous language speakers know of some method of contraception, whereas 99.2% of non-indigenous language speakers do. The gap becomes wider when it comes to understanding how contraceptive methods work since only 75.3% of indigenous speakers know how they work, compared to 95.9% of non-indigenous speakers. It may explain, in part, the low prevalence of contraceptive methods (24).

Due to the diverse containment measures adopted in response to the pandemic, the consequences generated are also diverse. We found more than double the possibility of not using contraceptives during the pandemic compared to the pre-pandemic period. The results of our study are in line with the trend estimated using a model to analyze the consequence of the covid-19 pandemic on access to contraceptives in Latin America and the Caribbean. When developing the model, considerations included the need to purchase contraceptives privately, the shortage of contraceptives in public services, the discontinuity of sexual and reproductive health services, and the reluctance of users to visit clinics for fear of infection during the pandemic (15). There is a need to explore further the reasons for low contraceptive use in everyday situations, not just during emergencies. The consequences of some factors on contraceptive use, such as indigenous status, should be considered for future health policies.

### Limitations

One notable advantage of this study is that it relied on primary data gathered through a questionnaire designed for this purpose. Moreover, the participants provided a comparison group by reporting their pregnancy experiences before the pandemic. While the authors recognize the potential for recall bias with a retrospective cross-over design, this is deemed less likely due to the non-traumatic nature of the questions asked. In Mexico, numerous indigenous groups speak distinct languages and reside in diverse communities with varying beliefs and perspectives, including reproductive health. Although our findings are based on a limited sample of participants from a specific community, they might be relevant for other communities sharing comparable characteristics.

## Conclusion

During the COVID-19 pandemic, indigenous women in the studied community used fewer contraceptive methods and did not use intrauterine devices. Additionally, there was a decline in the percentage of women using contraceptives. The results highlight the impact on indigenous populations and the difficulties they could face in accessing reproductive health services during health emergencies.

## Data availability statement

The original contributions presented in the study are included in the article/supplementary material, further inquiries can be directed to the corresponding author.

## Ethics statement

The studies involving human participants were reviewed and approved by The Ethics and Research Committee of the School of Medicine of the National Autonomous University of Mexico. Written informed consent to participate in this study was provided by the participant or the participants’ legal guardian/next of kin if they were underaged.

## Author contributions

All authors listed have made a substantial, direct, and intellectual contribution to the work and approved it for publication.

## Funding

This research project was funded by National Autonomous University of Mexico PAPIIT-DGAPA, grant number IA304521.

## Conflict of interest

The authors declare that the research was conducted in the absence of any commercial or financial relationships that could be construed as a potential conflict of interest.

## Publisher’s note

All claims expressed in this article are solely those of the authors and do not necessarily represent those of their affiliated organizations, or those of the publisher, the editors and the reviewers. Any product that may be evaluated in this article, or claim that may be made by its manufacturer, is not guaranteed or endorsed by the publisher.
